# Mycotoxins in Portuguese Agricultural Maize Fields and Dairy Farms

**DOI:** 10.3390/toxins16080335

**Published:** 2024-07-29

**Authors:** Marta Leite, Andreia Freitas, Jorge Barbosa, Fernando Ramos

**Affiliations:** 1 Faculty of Pharmacy, Health Science Campus, University of Coimbra, Azinhaga de Santa Comba, 3000-548 Coimbra, Portugal; marta.leite@iniav.pt; 2 National Institute for Agricultural and Veterinary Research (INIAV), Rua dos Lágidos, Lugar da Madalena, 4485-655 Vila do Conde, Portugal; andreia.freitas@iniav.pt; 3 Associated Laboratory for Green Chemistry (LAQV) of the Network of Chemistry and Technology (REQUIMTE), R. D. Manuel II, 4051-401 Porto, Portugal; jmsbarbosa@hotmail.com

**Keywords:** mycotoxins, maize, dairy farms, occurrence, food safety

## Abstract

Fungal and mycotoxin control at a primary stage in the food chain is crucial to maintaining the nutritional quality of animal feed. The control of fungal and mycotoxins is one of the essential points that a good biosecurity program must establish to ensure the safe feeding and protection of animal and human health. Acquiring a comprehensive understanding of the role of mycotoxins is vital to identifying breaches of this control and enabling the performance of proper risk assessments and accurate risk management strategies. This study focused on the identification of regulated and emerging mycotoxins in agricultural fields and dairy farms through an analytical methodology by ultra high-performance liquid chromatography coupled to tandem mass spectrometry (UHPLC-QTrap-MS/MS). This allowed us to identify a Portuguese mycotoxin profile in the maize value chain for the first time. Regarding our occurrence data, mycotoxins were identified in all samples, namely seeds, flowering plants, grain and forage at harvest, maize silage, and animal feed. FBs and ENNB were highly present in all stages of the production cycle. This work offers an initial insight into a full screening of regulated and emerging mycotoxins within an important agricultural commodity. The high occurrence of these compounds points to the need to perform occurrence surveys from an integrative perspective to protect consumers’ health, especially within food chains that provide various important staple foods worldwide.

## 1. Introduction

Maize (*Zea mays* L.) is a highly valuable agricultural and industrial product in worldwide food supplies. It is prone to being colonized by mycotoxigenic fungi and, consequently, contaminated by mycotoxins. This has severe negative impacts for farmers and livestock workers due to direct losses in crops, production profitability, animal health, and product safety [1]. Mycotoxins are toxic secondary metabolites that enter maize value chains in pre-harvest and post-harvest stages. Fumonisins (FBs), trichothecenes (TCTs), zearalenone (ZEA), aflatoxins (AFs), and ochratoxin (OTA) represent the main mycotoxins associated with the production cycle and storage [2]. Post-harvest maize is then used either for human consumption as grains or for animal consumption, also as grains, or as forage maize to be used as silage [3].

Animal diets are often a combination of several feed raw materials, mainly composed of maize silage and compound feed. Other ingredients, such as ensiled byproducts, are also used as feedstuffs [4]. Storage and pre-harvest conditions can lead to the contamination by mycotoxins of the individual components in animal feed. This contributes to the presence of multiple mycotoxins from different origins and, consequently, to the final total daily intake of these compounds by dairy animals [5,6]. The main mycotoxins found in maize silage, the main component of animal feed which is of special importance in animal nutrition, are *Fusarium* mycotoxins, including deoxynivalenol (DON), ZEA, FBs, nivalenol (NIV), fusaric acid (FUS A), and emerging mycotoxins, enniatin A (ENNA), enniatin B (ENNB), and beauvericin (BEA) [7]. ZEA and DON both occur in pre-harvest and post-harvest, being also characteristic of animal feed co-contamination patterns [8,9,10,11]. Nonetheless, the scarcity of studies on the single contribution of each component indicates that the current scenario needs further attention for the proper implementation of mitigation strategies.

Regulatory frameworks have been established for maximum and permitted levels of mycotoxins in different feed and food commodities due to their toxicity, with specific regulation regarding the latter [12,13,14]. These regulations only concern maize products intended for human or animal consumption and animal feed, specifically addressing aflatoxin B1 (AFB1), total AFs, DON, total FBs, OTA, T-2 and HT-2 toxins, and ZEA [1,15]. 

The development and implementation of mitigation strategies regarding the presence of mycotoxins in food chains is vital to ensure sustainable and safe food and feed production, though it is a great challenge to focus on whole value chains for assessments of mycotoxin profiles [16]. Our current study presents analytical data for 22 mycotoxins, including regulated, non-regulated, and emerging mycotoxigenic compounds: AFB1, aflatoxin B2 (AFB2), aflatoxin G1 (AFG1), aflatoxin G2 (AFG2), BEA, citrinin (CIT), DON, ENNA, ENNB, FB1, FB2, HT-2, mycophenolic acid (MPA), moniliformin (MON), NIV, OTA, penicillic acid (PA), patulin (PAT), T-2, tenuazonic acid (TEA), tentoxin (TTX) and ZEA. Work was performed on samples that are representative of maize cultivars intended for human consumption-products and animal feed and on dairy farms (grass and maize silage and complete animal feed). A total of 30 cultivars and 6 dairy farms were monitored and the corresponding samples were analyzed by a validated QuEChERS-based method, followed by liquid chromatography-mass spectrometry detection in order to obtain the characteristic occurrence patterns of mycotoxins in crucial stages of the maize value chain.

## 2. Results and Discussion

### 2.1. Mycotoxin Levels in Agricultural Maize Fields

A total of thirty agricultural maize fields were monitored for the presence of 22 mycotoxins at three stages of the growing maize production, namely sowing (28 samples), anthesis (30 aggregate samples of a total of 2150 plants), and harvesting (22 maize grain samples and 5 silage samples). The number of incidences and the percentage of positives, the mean, median, and maximum concentrations (µg kg^−1^), and the concentration range were calculated for all samples and are discussed individually in the subsections below. Overall, it was found that FBs and emerging mycotoxins, namely BEA and ENNB, were the most prevalent mycotoxins in agricultural fields throughout the various growing stages. High contents were found for several mycotoxins in the different samples, though in most samples, no exceeding values were found concerning the levels permitted by European regulation.

#### 2.1.1. Sowing Stage: Seeds

The quality of seeds is the basis for sustainable maize cultivation, and seed health is dependent on sustainable seed systems [17]. Seeds for sowing are basically grain maize harvested from agricultural fields to be used in the next production cycle. The main treatments for the storage of these products consist of fungicides and/or insecticides. Nonetheless, the treatment is directed at seed protection to protect emerging seedlings from soilborne fungal diseases and insect pests. Post-harvest mycotoxin content can, therefore, remain in the grains used as seeds in the first stage of crop cultivation. These toxins are not eliminated by traditional treatments, and contamination can occur during storage. In this work, seed samples (*n* = 28) corresponding to the seeding of 30 parcels were analyzed for the determination of 22 regulated, non-regulated, and emerging mycotoxins. The results are shown in Table 1.

From a total of 22 mycotoxins analyzed, only ten were found to be present at this stage of the maize growing production. The highest percentages of positives were found to be BEA and FB2, both at a frequency of 14%, followed by ENNB (11%). TTX and ZEA presented 7% frequencies in the 22-bulk sampling, while AFG2, ENNA, FB1, MON, and MPA were present in only 4% of the samples. In a study by Biemond et al. (2021), an overview of mycotoxigenic fungi in maize seed samples obtained from farmers, seed companies, and foundation seed producers was performed. In this study, the authors concluded that fungi producing FBs were of 100% infection frequency in all different seed commodities. Other mycotoxin-producing fungi, such as AFs, OTA and DON, presented frequencies higher than 50% [17]. 

High contents of FBs were also found in our study, namely 219.1 µg kg^−1^ in one sample for FB1, and an average of 382.2 µg kg^−1^ for FB2. One sample presented a maximum FB1 concentration of 1537.4 µg kg^−1^. This is nonetheless below the regulated value for this mycotoxin in products intended for animal feed, which ranges between 5000 to 60,000 µg kg^−1^ (a comparison to regulatory frameworks was done on the basis of products not intended for human consumption) [13]. A concentration of 285.6 µg kg^−1^ for AFG2 was also found in one seed sample. The regulated mycotoxin ZEA was equally present in two samples at concentrations of 2.1 and 7.3 µg kg^−1^, i.e., 1000 and 285-fold lower than the established guidance levels for this mycotoxin in products intended for animal feed. 

To the authors’ knowledge, this is the first time a study on maize seeds for sowing has been performed regarding mycotoxin determination. The present study notably considered a variety of toxic compounds not included in regulatory frameworks. A potential link between contaminated seeds and preharvest contamination is yet to be fully understood. Thus, the results obtained in this study corroborate the need for further studies on the quality and safety of grain intended for seeds, also foreseeing prospective soil contamination.

#### 2.1.2. Anthesis Stage: Whole-Plant Flowers

Different cropping factors, including seeding and harvest date, crop density (plants/hectare), seed treatment, crop rotations, cropping techniques (e.g., tillage or sowing), and environment (temperature and humidity), can lead to the contamination of cereal crops during their growing field production in the seedling to harvest stages [16]. To ensure limited post-harvest mycotoxin contamination, it is crucial to understand mycotoxin appearance and its relation to the maize growth stages. Recognizing that colonization by mycotoxin-producing fungi does not always result in mycotoxin presence is key for effective pre-harvest management. To understand pre-harvest contamination patterns concerning mycotoxin occurrence, a collection of samples was performed, as previously described. Briefly, 50 plants (less or equal to 2 hectares) and 100 plants (between 2 to 15 hectares) per field were harvested in the anthesis stage in the 30 study parcels (30 aggregate samples). Frequency and concentration data for the collected and analyzed samples are shown in Table 2.

At the stage of anthesis in maize cultivars, FBs presented the highest percentage of positives for regulated mycotoxins, namely, in 53 and 67% of the samples. The concentration values were also the highest for these mycotoxins, with FB1 concentrations ranging from 92.9 to 5988.6 µg kg^−1^. No DON was detectable in the 30 aggregate samples, though NIV was found in two samples with concentrations of 412.9 and 807.4 µg kg^−1^. The non-regulated mycotoxin TTX was found in 44% of the samples at very low concentrations, ranging between 3.2 and 9.9 µg kg^−1^. Regarding emerging mycotoxins, BEA, ENNB, and MON occurred in several samples at percentages of 33, 80, and 3%, respectively. Average concentrations of BEA and ENNB were of 78.3 and 21.0 µg kg^−1^, with maximum values of 261.3 and 150.6 µg kg^−1^, respectively. MON was found in one sample with a relatively high concentration compared to the data obtained (193.5 µg kg^−1^). Other mycotoxins were found in a lower number of samples and included the regulated mycotoxins AFB2 (7%), and AFG1 (13%), with maximum values of 3.4 and 123.8 µg kg^−1^, respectively.

In a previous study concerning the occurrence of mycotoxins in a Spanish maize production cycle for three consecutive years, the anthesis phase was also analyzed for regulated mycotoxins, namely, AFB1, OTA, FBs, HT-2 and T-2 toxins, DON, and ZEA [2]. Similar to our study, no AFB1, OTA, HT-2 and T-2 toxins, DON, or ZEA were found in this stage. On the other hand, detectable amounts of FB1 and FB2 were found at a concentration sum between 111.2 to 5902.9 µg kg^−1^, which corroborates our findings. A lack of data regarding this stage is an issue that needs to be overcome in order to promote strategies to reduce fungal development as well as mycotoxin production at this early stage of the maize production cycle.

#### 2.1.3. Harvested Maize: Grain and Forage

Maize is one of the most susceptible cereal crops to mycotoxigenic fungi and mycotoxin contamination. At harvest, the accumulation of mycotoxins through the growing cycle leads to the contamination of the final products for human and animal consumption. Even after processing and manufacturing procedures, these toxic compounds can persist, since some of them present high thermostability. In our study, a mycotoxin analysis was performed on 30 parcels corresponding to 27 samples, with 5 samples of maize forage intended for animal feed and 22 samples of maize grain (2 intended for the bakery industry, 6 intended for animal feed, and 14 as experimental fields). In Table 3, data obtained concerning these fields are presented in terms of positive samples (number and percentage), mean, median and maximum concentrations, and concentration ranges for the mycotoxins in analysis.

As in the previous stages, BEA, FB1, and FB2 presented high frequency values, with 67, 44, and 63%, respectively. Nonetheless, the highest frequency was found for MPA, which was positive in 21 samples (78%). ZEA was also found for the first time, at a frequency of 67% and a maximum concentration value of 1051.6 µg kg^−1^. Globally, the mycotoxin profile at harvest was more substantial in terms of the number of mycotoxins than in the other stages, with only AFG1, AFG2, HT-2, NIV, PAT, and T-2 not being identified in any of the samples. AFB1 and OTA were found at 0.8 and 9.1 µg kg^−1^ in only one sample each. Low frequencies of AFB2, DON, ENNA, PA, TEA, and TTX were also found, with values ranging from 11 to 15%. Concentration means of between 0.8 (AFB1) and 1582.4 (MPA) µg kg^−1^ were observed. Maximum values were found to be in a range of 5.9 (AFB2) to 5153.2 (BEA) µg kg^−1^, with the higher values attributed to forage maize (whole plant). All samples were below the permitted or guidance values established in European regulatory frameworks for all regulated mycotoxins.

The mycotoxic pattern found in the Portuguese maize fields in our study was in line with reports on this stage of the maize production cycle. These results confirm the importance of re-evaluating Good Agricultural Practices (GAP) in our country.

#### 2.1.4. Overview of the Presence of Mycotoxins in Maize Agricultural Production

**Figure 1:**
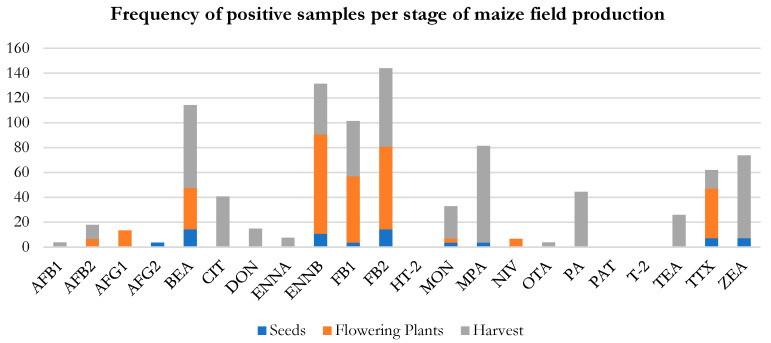
Frequency of positive samples in representative samples of the maize agricultural production: seeds, flowering plants, and at harvest (forage and grain).

Assessing value chains for mycotoxins is a challenge, though it gives an insight into how contaminations progress, allowing us to understand the crucial risk points in each stage of the growing process. In this work, a complete assessment of three important stages of the maize production cycle was carried out: sowing, anthesis, and harvesting. The results of frequency of positive samples and mycotoxin content (average) in seeds, flowering plants, grains, and forages are summarized in Figure 1 and Table 4, respectively, providing a complete overview of the data obtained throughout the maize production chain.

It was observed that most of the samples were contaminated at the end of the production system. ABF1, CIT, DON, OTA, PA, and TEA were only found at a later stage of the maize production cycle, though AFB1 and OTA were only found to be positive in one single sample. For statistical significance, a higher number of samples should be analyzed. AFB2, BEA, and MON were detected both in the flowering and harvesting stages, with 7 to 11%, 33 to 67%, and 3 to 33% increases in positives. In a study by Kamika, Ngbolua, and Tekere (2016), the occurrence of aflatoxin in maize throughout the food supply was also tested [18]. They concluded that as the supply chain progressed, the contaminated samples comprised 16 out of 50 pre-harvest samples, reaching 100% at harvest, with levels 300 times higher than the maximum established limit. Other mycotoxins were detected at both stages, namely ENNB, FB1, FB2, and TTX, though with higher percentages in the flowering stage. This can be explained by the field dimension versus the harvested plants fixed at 100 per field between 2 to 15 hectares. A sampling design that is proportional with the field size is of great importance for accurate results due to mycotoxin heterogenicity. However, this can pose problems in field management when applying these studies to agricultural producers.

### 2.2. Mycotoxin Levels in Dairy Farms

In dairy farm facilities, contamination by mycotoxins occurs in the storage of feed materials. This leads to a bulk of mycotoxins from different sources. Feed materials for animal feed consist mainly of maize silage, with other, different feed materials being added to its final composition (25 to 50%). This includes grass silage, wet maize grain silage, and/or maize flour, depending on the farm producer. Therefore, six dairy farms were monitored for the presence of 22 mycotoxins by collecting samples of complete animal feed and the corresponding feed materials (maize silage, grass silage, wet grain silage, and maize flour). The results for positive samples and mycotoxin contents in maize silage (*n* = 6), grass silage (*n* = 5), wet grain silage (*n* = 3), maize flour (*n* = 1), and complete animal feed (*n* = 6) from Portuguese dairy farms are shown in Table 5.

As reported before, the most frequent mycotoxins were FBs, ENNB, and BEA. The emerging mycotoxins presented the highest values in maize silage, maize flour, and complete feed (100% of positive samples). At lower frequencies, AFB2, ENNB, TTX, and ZEA were also found in the dairy farm samples analyzed. Pattern contamination of maize silage samples comprised AFB1 (17%), BEA (100%), ENNA (33%), ENNB (100%), FB1 and FB2 (67% each), TTX (17%), and ZEA (33%), while this profile in complete animal feed was AFB1 (17%), BEA (100%), ENNA (17%), ENNB (100%), FB1 and FB2 (67% and 50%, respectively), and TTX (33%). The main difference between these types of samples is the presence of ZEA in two samples of maize silage but no detected samples in animal feed. Grass silage (*n* = 5) was contaminated with BEA (20%), ENNA (20%), and ENNB (60%), while the sample of maize flour contained BEA and ENNB. Wet grain maize samples (*n* = 3) were contaminated with BEA, ENNB, FB1, FB2, and ZEA at a frequency of 33% for all mycotoxins.

It can be concluded that the main source of mycotoxins in complete animal feed is maize silage, with other materials presenting a low frequency or absence of positives. However, these feed materials still contribute to the final mycotoxin composition in animal feed. For example, high concentrations of mycotoxins can be found with wet grain silage, with a sample presenting a maximum of 3738.7 µg kg^−1^ for the emerging mycotoxin BEA. This notwithstanding, all regulated mycotoxins presented concentration values below those required by the European frameworks. Gruber-Dorninger, Jenkins, and Schatzmayr (2019), in a global study including analyses of feed and feed raw materials from 100 countries across the globe during a period from 2008 until 2017, identified FBs at percentage of 80%, with a maximum value of 218,883.0 µg kg^−1^ [19]. They also identified AFB1 at a frequency of 24%, and ZEA in 44% of the samples. Nonetheless, while numerous studies exist globally and on smaller scales for such facilities, they still predominantly focus on regulated mycotoxins. It is therefore important to acknowledge the role of emerging mycotoxins in such facilities, as corroborated by our study data.

## 3. Conclusions

This work provides insights into a multifaceted approach that considers both pre- and post-harvest contamination patterns in the maize value chain in response to the increasing frequency of non-regulated and emerging mycotoxins and their accumulation throughout the maize chain, targeting very different samples. Mycotoxins in maize is a known health hazard, both to animal and humans, that requires integrated approaches combining control measures for different climatic and socioeconomic regions and crops. The overall profile obtained for Portuguese maize agricultural production revealed a need to implement new strategies for agricultural and farming practices. Biosecurity plans and quality control schemes are crucial for the reduction and mitigation of such toxic compounds. However, the need to review these strategies to integrate mycotoxin decontamination is still a great challenge.

## 4. Materials and Methods

### 4.1. Sampling Plan Design

**Figure 2:**
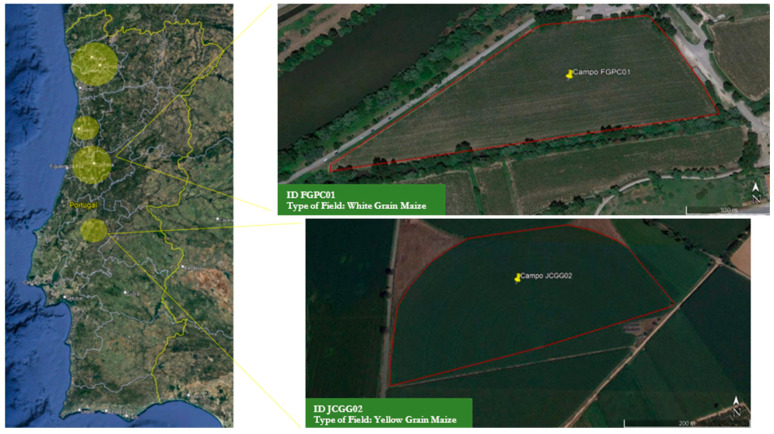
Schematic representation of sampling sites in Portugal, and field identification and delimitation for sampling design.

In 2019, a total of thirty agricultural maize fields were monitored for sample collection during the stages of (1) sowing, (2) flowering, and (3) harvesting. The selection of maize fields was based on a number of farms over the representative areas of maize production in Portugal, agricultural field size, maize type, and type of production (e.g., maize grain for human or animal consumption, and maize forage for animal consumption), distributed as follows: three fields in the north region of Portugal, two in the central coastal area, twelve in the inner center area, and three in the Ribatejo area (Figure 2). Sampling in agricultural maize fields was performed on-site on the corresponding days of each stage of maize growth. Seeds were collected from the reservoirs of the pneumatic seeding machines and harvested maize from the row maize picker sheller. Complete animal feed, maize silage, grass silage, and other feed materials were collected from the main Portuguese dairy region in the north of Portugal in the years of 2020 and 2021. Animal feed components were directly collected from silos, and the corresponding complete animal feed was collected after mixing in the Unifeed farm equipment. Samples were weighed in the field with portable weighing scales. Sterile bags were used to collect and store all collected samples.

Our sampling design plan was established according to Commission Regulation (EC) No 401/2006 and Commission Regulation (EC) No 519/2014 on the official control of the levels of mycotoxins in foodstuffs to guarantee the precision of data analyses regarding the determination of the mycotoxin content in each sample, due to the characteristic heterogeneity of mycotoxin distribution in any given lot [20,21]. In this respect, per farm, a total of 100 g of seeds per field, 1 kg of harvested maize (grain and forage) per field, and 1 kg of silage (grass and maize) and complete animal feed each were collected by taking samples from different sites on the lots. 

Regarding the collection of individual samples in agricultural fields at the growing stage, sampling plans are not described in the regulations. To guarantee an approximate representation analysis of mycotoxins in this stage, our protocol was established considering the agricultural field size as follows: for fields between 2 to 15 hectares, 100 samples were individually and randomly collected, covering the full area; for fields of less or equal to 2 hectares, 50 samples were collected. All samples were collected with appropriate sterile material and stored in sterile bags. To implement a rigorous sampling plan design, all agricultural fields were subjected to geospatial identification and area delimitation using satellite imagery from Google Earth Pro software version 7.3^®^ (Figure 2). Data on the width and length of each field, number and length of cultivation rows, and number of plants per row were collected from each farmer. Based on that, specific collection points (CP) were identified, and sampling was performed in each throughout the entire field areas, with five flowering plants being collected per CP. All collected incremental samples were combined per lot, and final aggregate samples were ground using appropriate grinders before undergoing the sample preparation protocol.

### 4.2. Sample Preparation and Mycotoxin Analysis

Mycotoxin determination was performed according to the validation method previously performed, which comprised the following compounds: AFB1, AFB2, AFG1, AFG2, BEA, CIT, DON, ENNA, ENNB, FB1, FB2, HT-2, MPA, MON, NIV, OTA, PA, PAT, T-2, TEA, TTX, and ZEA [22,23]. The criteria for each compound and the corresponding matrices, including limits of quantification (LOQ), were according to our previous work on method validation which followed Commission Regulation (EU) No 519/2014 for regulated mycotoxins and Commission Implementing Regulation (EU) 2021/808 for non-regulated mycotoxins [20,21,24]. Briefly, all final aggregate samples were completely and finely ground with sieves of 1- and 5-mm size on a Retsch mill (Düsseldorf, Germany). The freshly ground matrices were then weighed (2.0 ± 0.1 g for seeds and grains; 5.0 ± 0.1 g for flowering plants, forage, silage, and animal feed) into a 50-mL centrifuge tube, and 20 mL-volume of ACN:H_2_O (80:20, *v*/*v*) was added to each sample with further homogenization for 60 min at room temperature. QuEChERS protocol was applied, consisting of a mixture of 0.5 g of NaCl and 2.0 g of MgSO_4_ (1:4, *w*/*w*) for the salting-out step, and 150 mg C18 and 900 mg MgSO_4_ for the dSPE step on a 10 mL extract. The supernatant was collected and completely dried under nitrogen at a temperature of 40 °C. Reconstitution of the residue was performed by adding 500 µL of ACN 40% and filtering into HPLC vials. Injection was performed on a liquid chromatographic system coupled to a tandem mass detector (UHPLC-MS/MS) system with a total volume of 20 µL.

The UHPLC-MS/MS system for chromatographic separation and mass spectrometry determination was a UHPLC Nexera X2 Shimadzu system (AB Sciex, Foster City, CA, USA) coupled to a QTRAP 5500+ detector (AB Sciex, Foster City, CA, USA), with an electrospray ion source (ESI) operating in both positive and negative ion modes in a single run. Data acquisition and processing were performed in Multiple Reaction Monitoring (MRM), and MultiQuant^TM^ software, version 3.0.2. (AB Sciex, Foster City, CA, USA), respectively, according to the conditions previously established by Leite et al. [23]. The UHPLC system was a variable-volume autosampler with a refrigeration system, a binary pump, and a thermostatic column compartment with an analytical reverse-phase Gemini NX C18 110 Å 3.0 μm (100 × 2.0 mm i.d.), running at a flow rate of 0.2 mL min^−1^ with a mobile phase composition of (A) 0.1% formic acid and (B) acetonitrile. The gradient elution protocol was 95% A to 30% A (15 min), 30% A to 0% A (5 min, 2-min hold), and 0% A to 95% A (3 min). The autosampler and column compartment were maintained at 10 and 30 °C, respectively.

### 4.3. Descriptive Analysis

A descriptive data analysis was performed to calculate the absolute and relative frequencies of contaminated samples per total of analyzed samples. Samples with results lower than the LOD values were considered negative for contamination, while samples with values ranging between LOD and LOQ were assigned the corresponding LOQ values.

## Figures and Tables

**Table 1:** Mycotoxin data for maize seeds from the agricultural sowing stage (*n* = 28).

Mycotoxins	Nr. Positive Samples	% Positive Samples	Mean Concentration (µg kg^−1^)	Median Concentration (µg kg^−1^)	Maximum Value (µg kg^−1^)	Range (µg kg^−1^)
AFG2	1	4	285.6 *	-	-	-
BEA	4	14	127.6	111.2	280.2	17.8–280.2
ENNA	1	4	34.6 *	-	-	-
ENNB	3	11	14.4	14.5	14.6	14.1–14.6
FB1	1	4	219.1 *	-	-	-
FB2	4	14	382.2	13.63	1537.4	11.9–1537.4
MON	1	4	25.4 *	-	-	-
MPA	1	4	63.5 *	-	-	-
TTX	2	7	7.2	7.1	7.2	7.1–7.2
ZEA	2	7	4.7	4.7	7.3	2.1–7.3

* Single contaminated samples. AFG2—Aflatoxin G2; BEA—Beauvericin; ENNA—Enniatin A; ENNB—Enniatin B; FB1—Fumonisin B1; FB2—Fumonisin B2; MON—Moniliformin; MPA—Mycophenolic acid; TTX—Tentoxin; ZEA—Zearalenone.

**Table 2:** Mycotoxin data in flowering plant samples from Portuguese agricultural maize fields (*n* = 30).

Mycotoxins	Nr. Positive Samples	% Positive Samples	Mean Concentration (µg kg^−1^)	Median Concentration (µg kg^−1^)	Maximum Value (µg kg^−1^)	Range (µg kg^−1^)
AFB2	2	7	2.7	2.7	3.4	1.9–3.4
AFG1	4	13	90.1	95.9	123.8	45.0–123.8
BEA	10	33	78.3	39.8	261.3	21.7–261.3
ENNB	24	80	21.0	13.0	150.6	11.5–150.6
FB1	16	53	1487.2	426.7	5988.6	92.9–5988.6
FB2	20	67	363.8	255.0	1479.3	56.3–1479.3
MON	1	3	193.5 *	-	-	-
NIV	2	7	610.2	610.2	807.4	412.9–807.4
TTX	12	40	4.5	4.0	9.9	3.2–9.9

* Single contaminated samples. AFB2—Aflatoxin B2; AFG1—Aflatoxin G1; BEA—Beauvericin; ENNB—Enniatin B; FB1—Fumonisin B1; FB2—Fumonisin B2; MON—Moniliformin; NIV—Nivalenol; TTX—Tentoxin.

**Table 3:** Mycotoxin data in grain and forage samples from Portuguese agricultural fields at harvest (*n* = 27).

Mycotoxins	Nr. Positive Samples	% Positive Samples	Mean Concentration (µg kg^−1^)	Median Concentration (µg kg^−1^)	Maximum Value (µg kg^−1^)	Range (µg kg^−1^)
AFB1	1	4	0.8 *	-	-	-
AFB2	3	11	3.4	2.7	5.9	1.8–5.9
BEA	18	67	765.0	314.1	5153.2	15.2–5153.2
CIT	11	41	310.7	298.5	446.6	222.6–446.6
DON	4	15	191.0	172.8	383.2	35.3–383.2
ENNA	2	7	36.1	36.3	37.9	34.2–37.9
ENNB	11	41	76.0	42.1	478.9	13.8–478.9
FB1	12	44	376.5	46.0	3596.5	14.9–3596.5
FB2	17	63	197.8	197.2	350.0	25.6–350.0
MON	7	26	212.1	63.5	923.5	22.6–923.5
MPA	21	78	1582.4	1661.5	3254.5	53.3–3254.5
OTA	1	4	9.1 *	-	-	-
PA	3	11	20.7	20.7	24.3	15.5–24.3
TEA	4	15	48.1	49.1	88.0	6.2–88.0
TTX	4	15	8.8	9.0	10.3	7.4–10.3
ZEA	18	67	466.6	387.6	1051.6	10.7–1051.6

* Single contaminated samples. AFB1—Aflatoxin B1; AFB2—Aflatoxin B2; BEA—Beauvericin; CIT—Citrinin; DON—Deoxynivalenol; ENNA—Enniatin A; ENNB—Enniatin B; FB1—Fumonisin B1; FB2—Fumonisin B2; MON—Moniliformin; MPA—Mycophenolic acid; OTA—Ochratoxin; PA—Penicillic Acid; TEA—Tenuazonic acid; TTX—Tentoxin; ZEA—Zearalenone.

**Table 4:** Mycotoxin content (mean) in seeds, flowering plants, and at harvest (grain and forage).

Mycotoxins	Mean Mycotoxin Concentration (µg kg^−1^)		
	Seeds	Flowering Plants	Harvest
AFB1	<LOQ	<LOQ	0.8
AFB2	<LOQ	2.7	0.5
AFG1	<LOQ	90.1	<LOQ
AFG2	285.6	<LOQ	<LOQ
BEA	127.6	78.3	765.0
CIT	<LOQ	<LOQ	310.7
DON	<LOQ	<LOQ	191.0
ENNA	34.6	<LOQ	36.1
ENNB	14.4	21.0	76.0
FB1	219.1	1487.2	254.9
FB2	35.0	363.8	184.3
HT-2	<LOQ	<LOQ	<LOQ
MON	25.4	193.5	166.3
MPA	63.5	<LOQ	1582.4
NIV	<LOQ	610.2	<LOQ
OTA	<LOQ	<LOQ	9.1
PA	<LOQ	<LOQ	8.4
PAT	<LOQ	<LOQ	<LOQ
T-2	<LOQ	<LOQ	<LOQ
TEA	<LOQ	<LOQ	28.4
TTX	7.2	4.5	8.8
ZEA	0.4	<LOQ	466.6

AFB1—Aflatoxin B1; AFB2—Aflatoxin B2; AFG1—Aflatoxin G1; AFG2—Aflatoxin G2; BEA—Beauvericin; CIT—Citrinin; DON—Deoxynivalenol; ENNA—Enniatin A; ENNB—Enniatin B; FB1—Fumonisin B1; FB2—Fumonisin B2; LOQ—Limit of Quantification; MON—Moniliformin; MPA—Mycophenolic acid; NIV—Nivalenol; OTA—Ochratoxin; PA—Penicillic Acid; PAT—Patulin; TEA—Tenuazonic acid; TTX—Tentoxin; ZEA—Zearalenone.

**Table 5:** Mycotoxin data in samples from Portuguese dairy farms (*n* = 6).

Mycotoxins	Type of Samples	Nr. Samples	Nr. Positive Samples	% Positive Samples	Mean Conc. (µg kg^−1^)	Median Conc. (µg kg^−1^)	Max. Value (µg kg^−1^)	Range (µg kg^−1^)
AFB2	Maize silage	6	1	17	9.0	-	-	-
	Grass silage	5	0	0	-	-	-	-
	Wet grain silage	3	0	0	-	-	-	-
	Maize flour	1	0	0	-	-	-	-
	Animal feed	6	1	17	6.7	-	-	-
BEA	Maize silage	6	6	100	290.2	68.2	1001.6	36.6
	Grass silage	5	1	20	16.2 *	-	-	-
	Wet grain silage	3	1	33	3738.7 *	-	-	-
	Maize flour	1	1	100	20.6 *	-	-	-
	Animal feed	6	6	100	227.1	33.47	1026.5	21.3
ENNA	Maize silage	6	2	33	35.7	35.7	37.4	34.2
	Grass silage	5	1	20	37.4 *	-	-	-
	Wet grain silage	3	0	0	-	-	-	-
	Maize flour	1	0	0	-	-	-	-
	Animal feed	6	1	17	35.1 *	-	-	-
ENNB	Maize silage	6	6	100	77.1	16.4	285.1	14.3
	Grass silage	5	3	60	19.3	15.7	27.8	14.5
	Wet grain silage	3	1	33	113.6 *	-	-	-
	Maize flour	1	1	100	14.51 *	-	-	-
	Animal feed	6	6	100	67.6	16.3	271.9	14.6
FB1	Maize silage	6	4	67	26.6	25.9	39.0	13.6
	Grass silage	5	0	0	-	-	-	-
	Wet grain silage	3	1	33	228.3 *	-	-	-
	Maize flour	1	0	0	-	-	-	-
	Animal feed	6	4	67	28.0	15.4	53.1	15.4
FB2	Maize silage	6	4	67	45.3	38.8	77.9	25.9
	Grass silage	5	0	0	-	-	-	-
	Wet grain silage	3	1	33	65.9 *	-	-	-
	Maize flour	1	0	0	-	-	-	-
	Animal feed	6	3	50	45.4	34.6	74.6	27.1
TTX	Maize silage	6	1	17	10.2 *	-	-	-
	Grass silage	5	0	0	-	-	-	-
	Wet grain silage	3	0	0	-	-	-	-
	Maize flour	1	0	0	-	-	-	-
	Animal feed	6	2	33	10.3	10.3	11.8	9.0
ZEA	Maize silage	6	2	33	35.2	35.2	63.8	6.6
	Grass silage	5	0	0	-	-	-	-
	Wet grain silage	3	1	33	42.3 *	-	-	-
	Maize flour	1	0	0	-	-	-	-
	Animal feed	6	0	0	-	-	-	-

* Single contaminated samples. AFB2—Aflatoxin B2; BEA—Beauvericin; ENNA—Enniatin A; ENNB—Enniatin B; FB1—Fumonisin B1; FB2—Fumonisin B2; TTX—Tentoxin; ZEA—Zearalenone.

## Data Availability

The original contributions presented in the study are included in the article. Further inquiries can be directed to the corresponding author.
